# Linking sedentary behavior and mental distress in higher education: a cross-sectional study

**DOI:** 10.3389/fpsyg.2023.1205837

**Published:** 2023-08-01

**Authors:** Ana Belen Subiron-Valera, Beatriz Rodriguez-Roca, Estela Calatayud, Isabel Gomez-Soria, Elena Andrade-Gómez, Yolanda Marcen-Roman

**Affiliations:** ^1^Faculty of Health Sciences, University of Zaragoza, Zaragoza, Spain; ^2^Research group Sapienf (B53_23R), Zaragoza, Spain; ^3^Research Group Sector III Healthcare (GIIS081), Institute of Research of Aragón, Zaragoza, Spain; ^4^Institute for Health Research Aragón, Zaragoza, Spain; ^5^Department of Nursing, University of La Rioja, Logroño, La Rioja, Spain; ^6^Department of Human Anatomy and Histology, Faculty of Medicine, University of Zaragoza, Zaragoza, Spain

**Keywords:** anxiety, depression, mentally active behavior, mentally passive behavior, sedentary behavior, university students

## Abstract

**Background:**

Sedentary behavior among university students could negatively affect their mental health.

**Objective:**

The aim of this study was to examine the relationship of mental health (anxiety and depression) and sedentary behavior between gender in Health Degrees at the University of Zaragoza.

**Design:**

Cross-sectional descriptive study.

**Participants:**

Sample of 257 University students who completed an online questionnaire.

**Methods:**

Sedentary behavior was assessed with the SBQ questionnaire. Anxiety and depression were assessed with the GADS questionnaire. The Mann–Whitney U test and multiple linear regression models were used.

**Results:**

In comparison to men, female students with symptoms of anxiety spend more time in total engaged in sedentary behaviors (10.56 ± 4.83) vs. (7.8 ± 3.28; *p* < 0.001) and mentally-passive sedentary activities [2.24 (1.57) vs. 1.15 (0.90; *p* < 0.005)]. Female students at risk of depression also spend more hours engaged in mentally-passive sedentary behaviors in comparison to men (8.28 ± 50.70 vs. 1.27 ± 1.02; *p* = 0.009).

**Conclusion:**

Female students at risk of anxiety and/or depression spend more time engaged in sedentary activities in comparison to male students. The risk of anxiety and depression is associated with the total number of hours a day spent engaged in sedentary behaviors and with mentally passive behaviors, but not mentally active behaviors.

## 1. Introduction

Time spent at university is critical for students to establish a lifestyle for adult life (Lee and Kim, 2018), based on continuous changes where healthy habits and future adult patterns are developed (World Health Organization, 2018). Despite this, sedentary behavior (SB) among higher education students is at alarming levels worldwide and students’ commitments often lead them to increase the number of waking hours spent in sitting activities (Nelson et al., 2008), such as studying and attending seminars (Cotten and Prapavessis, 2016). This situation involves minimal energy expenditure [≤1.5 metabolic equivalents (METs)] and mainly time spent in sitting, reclining or lying activities (Tremblay et al., 2017). It is important to note that SB is not the same as not being physically active. Both situations can coexist together or separately and the negative effects caused by SB cannot always be neutralized by following the current recommendations of 30 min of physical activity per day (Dogra and Stathokostas, 2012; World Health Organization, 2018).

Regarding gender, it should be noted that the sample of health science students in this study are female, as students studying medicine or health sciences are predominantly female (Wörfel et al., 2016). On the association between gender and physical activity, there are currently discrepancies. On the one hand, some studies report that gender is not a variable that reports differences in sedentary behavior in the 18–29 age groups (Wallmann-Sperlich et al., 2013; Edelmann et al., 2022), nor in university students (Castro et al., 2020). On the other hand, there are studies that conclude that university women are more inactive compared to men. For example, a study conducted at the University of Japan concluded that a higher percentage of women did not engage in physical activity compared to men (García Puello and Herazo Beltrán, 2015); at a university in Chile, they also obtained the same conclusions (Morales et al., 2013).

Regarding the degree type and its relationship with a sedentary lifestyle, we found that there are university degrees in which students perform a higher level of physical activity compared to the rest of the degrees, such as all those related to the world of sport, such as a degree in physical education, physical activity and sport or sports training technology (Farinola et al., 2012). For degrees related to health sciences, only 42.5% (Castro Cuesta et al., 2014) engage in physical exercise and spend an average of 8.3 h a day (self-reported) on SB (Musaiger et al., 2017; Mussi et al., 2017).

SB has been found to have negative health effects, such as premature death as well as chronic and cardiovascular diseases (Patterson et al., 2018). In addition, SB may have an impact on mental health and the risk of developing anxiety or depression may increase (Primack et al., 2009; Teychenne et al., 2015) independently of physical activity (Ministerio de Salud, 2017; Jiang et al., 2020). Nowadays, depression is known to be the most common illness in university students (Liu et al., 2019), and an association between SB and anxiety or depression can be considered. Although studies in university students in Australia concluded that SB was not correlated with mental health (Rebar et al., 2015). On the other hand, a study in Spanish university students concluded that those students who spent more than 42 h a week sitting had a 31% higher risk of mental disorders (Sanchez-Villegas et al., 2008). Furthermore, a meta-analysis concluded that SB increases the risk of anxiety and depression (Zhai et al., 2015). Similarly, there are similar findings on associations between SB, anxiety, depression and gender, where anxiety is associated with women (Al-Qaisy, 2011) and depression is commonly associated with men (Brunet et al., 2014).

Research on SB is based on the analysis of several domains (such as screen time or transportation), but most studies have focused on a single domain or on the total amount of time (Wilmot et al., 2012), which makes it difficult to analyze correlations between several domains (Chastin et al., 2015). Recent studies have classified SB into two categories according to its cognitive stimulation: mentally-active SB and mentally-passive SB (Hallgren et al., 2020; Werneck et al., 2021). As mentioned above, it is important to acquire a healthy lifestyle during the university period and to continue it through to adulthood. Therefore, we conducted a cross-sectional study on a sample of university health science students. The aim of this research was (a) to describe the socio-demographic characteristics in a sample of university health science students in Zaragoza (Spain); (b) to examine whether anxiety or depression is associated with sitting time (during weekdays and weekends) according to gender. In addition, we would like to analyze whether there is any difference between how mentally active or passive SB influences the risk of developing anxiety or depression.

## 2. Materials and methods

### 2.1. Sampling and data collection

We carried out a quantitative study with a cross-sectional design. Our target population were all students from three particular degrees in the Health Sciences Faculty of the University of Zaragoza (Nursing, Physiotherapy, and Occupational Therapy). A sample size of 257 students was estimated by taking a 4.66% error margin for proportions for frequency of results on these scales, as calculated by the EPIDAT 4.2 program. A maximum margin for error of 5% was recommended. University students who did not answer any of the questions in the anxiety or depression questionnaire were not included in the corresponding part of the study.

An online questionnaire was designed and distributed to students of health sciences in March 2022 via a web link using the Moodle platform. All participants were completely anonymous and voluntary. The inclusion criteria for taking part in the study included: individuals studying at least on one of the three health science degrees, and individuals who agreed to the conditions of the study and gave informed consent to participate. Participants who could neither speak nor understand Spanish and who had not answered all the questions in the survey were excluded.

Participants were also invited to complete a sociodemographic questionnaire with the aim of describing the characteristics of our sample including the following variables: gender (male/female), age, degree (nursing, physiotherapy, occupational therapy), place of residence (urban/rural) and country of birth (Spain/elsewhere).

This study was performed in accordance with the Declaration of Helsinki. Data was processed confidentially in line with the Spanish Organic Law LOPD 03/2018 on Personal Data Protection. The Committee of Research Ethics of the Spanish Autonomous Aragón Community (CEICA) gave its approval before the study began (C.P.-C.I. PI22/060). The manuscript was written in accordance with the Strengthening the Reporting of Observational Studies in Epidemiology (STROBE) protocol (Elm et al., 2007).

### 2.2. Instruments

In order to gain knowledge of the sedentary behavior of students, the SBQ questionnaire (Rosenberg et al., 2010) and the Goldberg Anxiety and Depression scale (GADS; Goldberg et al., 1988; Molina et al., 2006) were used.

Participants were asked to self-report their daily sedentary time using the sedentary behavior questionnaire (SBQ) and comprised 12 items. Participants selected how long they spent per day engaged in various sedentary activities such as watching TV, reading a book, etc. The daily average sedentary behavior was calculated for weekdays and weekends separately. The SBQ questionnaire is a valid tool for assessing SB (Sansano-Nadal et al., 2022) and has a good internal consistency (α ranges from 0.48 to 0.93) as well as excellent reliability (r = 0.64 to 0.90 for weekdays and r = 0.51 to 0.93 for weekend days; Rosenberg et al., 2010).

Anxiety and depression were measured with the abbreviated GADS, a self-reported scale developed by Goldberg in the general population (Goldberg et al., 1988), by examining four basic psychiatric areas: depression, anxiety, social anxiety disorder, and hypochondria. The questionnaire consists of 18 items. This instrument has been previously validated and its applicability has been demonstrated. The cut off points were equal to or higher than 4 for the anxiety score, and equal to or higher than 2 for depression. Sensitivity (83.0%) and specificity (81.8%) give a 95% positive predictive value (Goldberg et al., 1988).

### 2.3. Data analysis

First of all, the Mahalanobis distance was carried out. Afterwards, a descriptive analysis was performed with frequencies and percentages for the qualitative variables, and with the mean and standard deviation (SD) for the quantitative variables. The normality of the variables was explored using the Kolmogorov–Smirnov test. To compare the variables, the Mann–Whitney *U*-test was used to examine differences between anxiety and depression among gender and continuous variables: age and sitting time during weekdays as well as weekends engaged in mentally active or passive SB. The associations between anxiety, depression, and SB time—the time during weekdays and weekends spent engaged in mentally active and passive SB-were analyzed through multiple model regression for each outcome with age, gender, degree serving as covariates. The significance level of all the analyses was set at *p* ≤ 0.05. The quantitative data was analyzed using SPSS, version 26 (IBM, Corp., Chicago, IL, United States).

## 3. Results

A total sample of 257 students of Health Sciences participated in this study [38.5% Nursing degree, 24.1% Physiotherapy degree and 37.4% OT (occupational therapy) degree]. The mean age of participants was 20.48 years (SD 4.88) and 78.2% were female. The majority were born in Spain (99.2%) and 93.7% lived in an urban area. The 74.7% of the students were not at risk of anxiety and the 65.8% were not at risk of depression (Table 1).

**Table 1:** Characteristics of the overall sample.

Variables	Total (*n* = 257)
	Mean (SD)
Age (years)	20.48 (4.88)
	**Frequencies (%)**
**Gender**	
Male	56 (21.8%)
Female	201 (78.2%)
**Country**	
Spain	255 (99.2%)
Elsewhere	2 (0.8%)
**Place of residence**	
Urban	240 (93.7%)
Rural	17 (6.3%)
**Degree**	
Nursing	99 (38.5%)
Physiotherapy	62 (24.1%)
Occupational therapy	96 (37.4%)
**Risk of anxiety (GADS)**	
Yes	65 (25.3%)
No	190 (73.9%)
Absent	2 (0.8%)
**Risk of depression (GADS)**	
Yes	88 (34.2%)
No	165 (64.2%)
Absent	4 (1.6%)

Gender differences were observed in students at risk of anxiety. Women who described themselves as suffering from a risk of anxiety displayed higher levels of sedentary behavior in comparison to men. This difference is particularly apparent during daytime hours when they attend lectures; sedentary behavior during the week was 51.63 (26.51) among women compared with 35.86 (16.06) in men (*p* < 0.001); while the total sedentary behavior was 10.56 (4.83) among women with anxiety, higher than the 8.7 (3.28) reported for men (*p* < 0.001).

In general, women with anxiety show higher levels of sedentary behavior, but it was primarily observed that women dedicated more time to mentally-active sedentary behavior than men, during weekdays 9.63 (7.05) vs. 5.05 (4.00; *p* < 0.001) and during the weekend 6.07 (4.88) vs. 3.03 (2.87; *p* < 0.001).

Equally, women were shown to have higher levels of total passive sedentary behavior, spending more time in general on this type of activity 2.24 (1.57) vs. 1.15 (0.9; *p* < 0.004). However, no gender differences were found to exist in relation to levels of passive sedentary behavior during the weekend (Table 2).

**Table 2:** Relationship between anxiety and sedentary behavior by gender.

SBQ questionnaire	Risk of anxiety				No risk of anxiety				Overall	
	Male (*n* = 19)	Female (*n* = 46)	Overall (*n* = 65)	*p*	Male (*n* = 36)	Female (*n* = 154)	Overall (*n* = 190)	*p*	Overall (*n* = 255)	*p*
	Mean (SD)	Mean (SD)	Mean (SD)		Mean (SD)	Mean (SD)	Mean (SD)		Mean (SD)	
**SB during week days**										
Sitting time (hours/week)	35.86 (16.06)	51.63 (26.51)	47.02 (25.88)	0.005^*^	50.56 (29.33)	53.4 (23.7)	52.86 (24.8)	0.214	51.37 (24.90)	0.026^*^
Mentally-active SB (hours/day)	5.05 (4.00)	9.63 (7.05)	8.29 (6.62)	0.003^*^	8.32 (7.00)	9.49 (5.59)	9.26 (5.88)	0.067	9.01 (6.08)	0.103
Mentally-passive SB (hours/day)	30.36 (3.65)	42 (6.51)	38.73 (5.47)	0.008^*^	42.23 (5.37)	43.91 (6.43)	43.5 (5.75)	0.086	40.02(5.25)	0.103
**SB during weekends**										
Sitting time (hours/weekend)	18.71 (8.52)	22.26 (8.66)	21.22 (8.71)	0.087	23.6 (13.65)	24.6 (10.33)	24.41 (11.01)	0.187	23.60(10.54)	0.028^*^
Mentally-active SB (hours/day)	3.03 (2.87)	6.07 (4.88)	5.18 (4.59)	0.018^*^	5.36 (5.56)	6.01 (4.59)	5.89 (4.77)	0.219	5.70(4.72)	0.288
Mentally-passive SB (hours/day)	15.68 (7.6)	16.2 (6.96)	16.05 (7.10)	0.788	18.42 (10.56)	18.59 (9.44)	18.56 (9.63)	0.715	17.91 (9.10)	0.080
**Total SB**										
Total SB (hours/day)	7.8 (3.28)	10.56 (4.83)	9.75 (4.58)	0.007^*^	10.59 (6.02)	11.14 (4.56)	11.04 (4.86)	0.155	10.71 (4.81)	0.009^*^
Total mentally-active SB (hours/day)	2.96 (1.26)	3.69 (1.64)	3.48 (1.57)	0.056	3.82 (2.15)	4.02 (1.76)	3.98 (1.83)	0.202	3.74 (26.20)	0.067
Total mentally-passive SB (hours/day)	1.15 (0.90)	2.24 (1.57)	1.92 (1.49)	0.004^*^	2 (1.77)	4.93 (3.69)	4.37 (3.03)	0.067	4.37 (30.34)	0.014^*^

*p*-value < 0.005^*^. Mann–Whitney *U*-test.

Nevertheless, gender differences were observed in students at risk of depression. Women who described themselves as feeling depressed spend more time engaged in sedentary behaviors in comparison to men. This is shown in the results for sedentarism during the week 50.9 (27.11) vs. 36.06 (16.54; *p* < 0.001) and total sedentarism 10.49 (5.21) vs. 7.78 (3.36; *p* = 0.010). It was also at higher levels among women engaged in active 9.11 (6.70) vs. 5.22 (3.76; *p* = 0.011) and passive 41.79 (5.32) vs. 30.84 (4.75; *p* = 0.011) sedentary behaviors during the week. There was no difference during the weekend either, where women were shown to have higher levels of active sedentary behavior than men on Saturdays and Sundays 5.79 (4.60) vs. 3 (3.03; *p* = 0.016).

In comparison to students who did not describe symptoms of depression in the GADS questionnaire, sedentary behaviors were similar in both genders and there were no significant differences (Table 3).

**Table 3:** Characteristics of the sedentary behaviors of the overall sample according to the risk of developing depression by gender.

SBQ questionnaire	Risk of depression				No risk of depression				Overall	
	Male (*n* = 20)	Female (*n* = 68)	Overall (*n* = 88)	*p*	Male (*n* = 35)	Female (*n* = 130)	Overall (*n* = 165)	*p*	Overall (*n* = 253)	*p*
	Mean (SD)	Mean (SD)	Mean (SD)		Mean (SD)	Mean (SD)	Mean (SD)		Mean (SD)	
**SB during weekdays**										
Sitting time (hours/week)	36.06 (16.54)	50.9 (27.11)	47.53 (25.78)	0.008^*^	52.5 (29.12)	54.4 (22.79)	54 (24.19)	0.277	51.74 (24.89)	0.010^*^
Mentally-active SB (hours/day)	5.22 (3.76)	9.11 (6.70)	8.22 (6.35)	0.011^*^	8.66 (7.09)	9.78 (5.56)	9.55 (5.91)	0.101	9.08 (6.08)	0.032^*^
Mentally-passive-SB (hours/day)	30.84 (4.75)	41.79 (5.32)	39.30 (5.87)	0.012^*^	43.84 (5.01)	44.62 (5.36)	44.45 (5.63)	0.123	42.25 (5.23)	0.032^*^
**SB during weekends**										
Sitting time (hours/weekend)	18.38 (8.62)	22.56 (10.80)	21.61 (10.45)	0.065	24.73 (13.43)	24.93 (9.56)	24.89 (10.45)	0.365	23.75 (10.54)	0.004^*^
Mentally-active SB (hours/day)	3 (3.03)	5.79 (4.60)	5.18 (4.45)	0.016^*^	5.4 (5.49)	6.14 (4.7)	5.98 (4.87)	0.193	3.77 (26.30)	0.239
Mentally-passive SB (hours/day)	15.57 (7.16)	16.76 (8.94)	16.49 (8.54)	0.911	19.33 (10.82)	18.79 (8.97)	18.91 (9.36)	0.888	18.06 (9.14)	0.033^*^
**Total SB**										
Total SB (hours/day)	7.78 (3.36)	10.49 (5.21)	9.88 (4.97)	0.010^*^	11.03 (5.96)	11.33 (4.28)	11.27 (4.67)	0.202	10.78 (4.81)	0.005^*^
Total mentally-active SB (hours/day)	2.97 (1.32)	3.72 (1.91)	3.55 (1.82)	0.052	4 (2.14)	4.08 (1.63)	4.06 (1.74)	0.307	4.34 (16.10)	0.087
Total mentally-passive SB (hours/day)	1.27 (1.02)	8.28 (50.70)	6.69 (44.59)	0.009^*^	2.01 (1.75)	2.28 (1.21)	2.22 (1.34)	0.073	5.27(25.18)	0.006^*^

*p*-value < 0.005^*^. Mann–Whitney *U*-test.

Table 4 shows the multiple linear regression models with daily SB (measured using the SBQ questionnaire) and covariates (anxiety, depression, degree, age, and gender). Depression was associated with mentally-active sitting time (R^2^ = 0.006, *p* = 0.037). However, no SB was associated with anxiety or gender. Degree was associated with sitting during weekdays (R^2^ = 0.046, *p* = 0.034), sitting time during weekends (R^2^ = 0.049, *p* = 0.009), total sitting time (R^2^ = 0.051, *p* = 0.016), and mentally passive sitting time (R^2^ = 0.042, *p* = 0.021). Age only was associated with sitting time during weekdays (R^2^ = 0.046, *p* = 0.049). The 95% of CI was applied (Table 5).

**Table 4:** Multiple linear regression model adjusted for anxiety, depression, degree, age and gender.

	Adj R^2^	Anxiety			Depression			Degree			Age			Gender		
		Mean square	F	*p*-value	Mean square	F	*p*-value	Mean square	F	*p*-value	Mean square	F	*p*-value	Mean square	F	*p*-value
Sitting weekdays	0.046	516.096	0.857	0.356	607.776	1.009	0.316	2066.279	3.430	0.034	2358.786	3.915	*0.049*	882.697	1.465	0.227
Sitting weekends	0.049	187.950	1.754	0.187	126.378	1.179	0.279	516.417	4.817	0.009	43.839	0.409	0.523	5.467	0.051	0.822
Total sitting time	0.051	27.080	1.210	0.272	26.295	1.175	0.280	94.737	4.233	0.016	62.159	2.777	0.097	20.961	0.937	0.334
Mentally active sitting time	0.006	1703.786	2.381	0.124	3161.460	4.417	0.037*	308.293	0.431	0.651	58.536	0.082	0.775	130.696	0.183	0.670
-Mentally passive sitting time	0.042	4.874	1.582	0.210	3.012	0.978	0.324	12.065	3.917	0.021	4.575	1.485	0.224	1.398	0.454	0.501

**Table 5:** Reliability of self-administered questionnaires.

Questionnaires	Alfa Cronbach
SBQ	0.831
GADS	0.853

## 4. Discussion

Through this study we were able to determine that gender influences the time spent in sedentary activities, being higher in women than in men. Within sedentary activities we concluded that there is a higher risk of developing anxiety and depression when spending time in mentally-passive sedentary activities such as watching television compared to mentally-active ones such as reading. The transition from secondary school to university education leads to a change in habits and lifestyles, which may encourage high-risk behaviors (Maselli et al., 2018). In the case of the student population in question, school days usually require sitting for prolonged periods of time.

Moreover, sedentary behavior is increasingly prevalent in the world population (Peterson et al., 2018), more specifically in Europe (Sjöström et al., 2006; Gerovasili et al., 2015). Similarly, Castro et al. (2020) show that university students spend more than 7.29 h per day doing sedentary activities and Mussi et al. (2017) indicate an average of 8.3 h per day. This data is consistent with the results obtained in our study in which 65% of students spend more than 9.75 h in situations involving sedentary behaviors.

In general, there are alarming figures on sedentary time among university students. More than half of all university students are sedentary, similar data reveals national and international studies, in which it is reported that most university students do little or no physical activity (García Puello and Herazo Beltrán, 2015; Práxedes et al., 2016). And approximately three out of every 10 individuals aged 15 years or older, around 1.5 billion people, do not meet current physical activity recommendations (Hallal et al., 2012).

The trend toward SBs has increased over the last few years, with the proportion of women in particular increasing (Verdot et al., 2022). In adolescence, studies show that, during and after puberty, girls perform worse than before in almost all fitness tests, as this period is characterized by an increase in sexual dimorphism in terms of physical fitness (Witkowski et al., 2018).

Focusing on gender, we find that the findings of this study correlate with those described by Elizondo and Guillen, in that women have a more sedentary lifestyle, while men have a slightly higher level of physical activity (Elizondo-Armendáriz et al., 2005). Several studies have concluded that physical inactivity is associated with gender, with women being more sedentary (Farinola and Bazán, 2011). This could be due, on the one hand, to the intrinsic motivation or qualities that each gender experiences or is able to develop by engaging in some form of physical activity and not remaining in sedentary postures. For example, studies on active individuals conclude that men excel in self-efficacy, solution seeking and goal orientation and that women develop greater resilience associated with physical activity (Patsiaouras, 2021). This could be one of the reasons that encourage male students to be physically active, and it could also influence physical activity practice that males prefer to exercise with peers while females prefer to exercise alone (Alkatan et al., 2021).

There is no consensus on whether the chosen university degree influences SB. On the one hand, authors such as (Nowak et al., 2019), indicate that students in physical health-related fields of study (physiotherapy) are more interested in physical activities during their free time, in the same way that students related to the medical field are more active than the general population of students of the same age range (Farinola and Bazán, 2011; Rangel Caballero et al., 2015; Nowak et al., 2019; Edelmann et al., 2022). In contrast, as reported by Stamatakis et al. (2019), health science students have high levels of sedentary behavior ≥ 8 h/day of total sitting time.

Although it may seem contradictory, it has been shown that people with anxiety have more sedentary behaviors, especially passive ones. It is also interesting to note that a higher percentage of women suffer from these disorders than men (McLean and Anderson, 2009). Women with anxiety have in general a more sedentary behavior than men, and this is statistically significant during weekdays, Monday to Friday. In contrast, a study in adults (Bennie et al., 2016) reports that men spend more time engaged in sedentary activities, and other studies find that sedentary behavior in men is characterized by more time spent on digital activities in general, while women spend that time studying (Lacy et al., 2012; Carballo-Fazanes et al., 2020). In terms of depressive disorders, authors such as Zhai et al. (2015), Madhav et al. (2017), and Zhang et al. (2022) indicate that high levels of sedentary lifestyles are associated with increased depression. In their adult population study, Kilpatrick et al. (2013) suggest that reducing the amount of time spent sitting in the world may be beneficial for mental health, finding an association between sedentary work and intermediate levels of psychological distress, independent of leisure-time physical activity. It is already known from previous research that physical activity has been associated with lower levels of anxiety in the general population (Rebar et al., 2015).

Importantly, Hallgren et al. (2020) concluded that not all sedentary activities have the same negative impact on health; several activities involve being mentally-active, such as reading, talking to others, studying, playing an instrument, performing manual tasks or driving, and this has a different effect on health compared to mentally-passive activities such as watching TV, for example (Hallgren et al., 2020). In addition, it is known that learning via computer or phone should not replace outdoor activities (Witkowski et al., 2018).

Sedentary behaviors during the week have an impact on how leisure time is spent on weekends (Arumi-Prat et al., 2022). Although one would expect students to spend their time at weekends in active leisure, engaging in activities related to movement and physical activity, it has been shown that they continue to engage in mentally-passive sedentary behaviors, especially females.

Time spent being sedentary is related to the likelihood of chronic diseases, whereas, in contrast, mentally-active behaviors can provide stimulation, become a protective activity, and promote healthy cognition other benefits that cannot be achieved by mentally-passive behaviors (Hallgren et al., 2018, 2020; Werneck et al., 2021). Passive domains such as television viewing (Werneck et al., 2018) and screen time (Zhai et al., 2015; Madhav et al., 2017; Zhou et al., 2021) are associated with higher levels of depressive symptomatology.

It would be important to keep in mind that the time spent as a student should be a time of learning, contact and discovery of a healthy lifestyle, and not only to prepare for the world of work that awaits them. In tune with this, Witkowski et al. indicate that athletes and even coaches of different sport modalities agree that an athlete’s personality can be enriched through training, sport achievements, with psychosomatic and educational impact (Witkowski et al., 2016).

As a basic goal, students should try to prevent sedentary habits from being sustained over time and leading to physical symptoms and diseases implicated in certain co-morbidities in later life (Wilmot et al., 2012; Patterson et al., 2018; Bailey et al., 2022). To this end, monitoring sedentary behaviors is essential to accurately assess the situation, identify needs and establish actions to be taken (World Health Organization, 2018).

Our study has limitations that could represent a selection bias as it is an observational study and all subjects participated voluntarily in it. Another limitation was that this study was conducted with a university population related to health and patient care, so the extrapolation of these results cannot be directly applied to all university students or to the general population, and they are specific to the time in which it was conducted, when Covid was still spoken about and felt.

## 5. Conclusion

Sedentary behaviors are elevated in female university students compared to males. Prolonged sitting is a potentially modifiable behavior, which should be modified because of its adverse implications for overall physical and psychological health. Furthermore, there is a significant association between prolonged sedentary behavior and the risk of developing anxiety and depression, being more pronounced among women. Among all sedentary activities, mentally-passive activities are associated with a higher risk of developing anxiety and depression than mentally-active ones. Although further research is needed over time before extrapolating to other university students, this data contributes to the emerging literature investigating the health outcomes associated with sedentary behaviors.

## Data availability statement

The raw data supporting the conclusions of this article will be made available by the authors, without undue reservation.

## Ethics statement

The studies involving human participants were reviewed and approved by Committee of Research Ethics of the Spanish Autonomous Aragón Community (CEICA). The patients/participants provided their written informed consent to participate in this study.

## Author contributions

YM-R, BR-R, and AS-V developed the protocol, monitored the data collection, performed the analyses, and interpreted the data. EC, EA-G, and IG-S drafted the manuscript. EC, BR-R, YM-R, and AS-V refined the definition of the indicators and contributed to the interpretation of the results. All authors contributed to the article and approved the submitted version.

## Conflict of interest

The authors declare that the research was conducted in the absence of any commercial or financial relationships that could be construed as a potential conflict of interest.

## Publisher’s note

All claims expressed in this article are solely those of the authors and do not necessarily represent those of their affiliated organizations, or those of the publisher, the editors and the reviewers. Any product that may be evaluated in this article, or claim that may be made by its manufacturer, is not guaranteed or endorsed by the publisher.
